# Community-derived recommendations for healthcare systems and medical students to support people who are houseless in Portland, Oregon: a mixed-methods study

**DOI:** 10.1186/s12889-020-09444-4

**Published:** 2020-09-02

**Authors:** Caroline King, Cameron Fisher, Jacob Johnson, Arum Chun, David Bangsberg, Paula Carder

**Affiliations:** 1 School of Medicine, Oregon Health & Science University, Portland, OR USA; 2 School of Public Health, Oregon Health & Science University-Portland State University, Portland, OR USA

**Keywords:** Mental health, Medical students, Homeless persons, Housing, Social stigma, Qualitative research, Substance-related disorders

## Abstract

**Background:**

People who are houseless (also referred to as homeless) perceive high stigma in healthcare settings, and face disproportionate disparities in morbidity and mortality versus people who are housed. Medical students and the training institutions they are a part of play important roles in advocating for the needs of this community. The objective of this study was to understand perceptions of how medical students and institutions can meet needs of the self-identified needs of the houseless community.

**Methods:**

Between February and May 2018, medical students conducted mixed-methods surveys with semi-structured qualitative interview guides at two community-based organizations that serve people who are houseless in Portland, Oregon. Medical students approach guests at both locations to ascertain interest in participating in the study. Qualitative data were analyzed using thematic analysis rooted in an inductive process.

**Results:**

We enrolled 38 participants in this study. Most participants were male (73.7%), white (78.9%), and had been houseless for over a year at the time of interview (65.8%). Qualitative themes describe care experiences among people with mental health and substance use disorders, and roles for medical students and health-care institutions. Specifically, people who are houseless want medical students to 1) listen to and believe them, 2) work to destigmatize houselessness, 3) engage in diverse clinical experiences, and 4) advocate for change at the institutional level. Participants asked healthcare institutions to use their power to change laws that criminalize substance use and houselessness, and build healthcare systems that take better care of people with addiction and mental health conditions.

**Conclusions:**

Medical students, and the institutions they are a part of, should seek to reduce stigma against people who are houseless in medical systems. Additionally, institutions should change their approaches to healthcare delivery and advocacy to better support the health of people who are houseless.

## Background

Over a half a million people are houseless (also referred to as homeless) on any given night in the United States [1]. People who are houseless are three to four times more likely to die than the average population, and the average life expectancy for a person who is houseless in the United States is between 42 and 52 years, whereas average life expectancy is nearly 35 years longer for the general population [2]. To address these disparities, much research has supported “housing as health”, citing emerging areas of research that have identified links between housing and health outcomes, including decreasing health utilization and improving self-reported mental and physical health [3, 4]. Importantly, a comparison of patients who were recently houseless versus currently houseless showed recently houseless patients were less likely to die drug or alcohol or hypothermia related causes than houseless controls, suggesting that persistent houselessness is an independent risk factor for worse health outcomes [5].

People who are houseless experience higher rates of alcohol, drug, and psychiatric comorbidities compared with people who are housed [6, 7], as well as higher rates of physical health conditions [8]. Healthcare seeking is common, though often delayed, among people who are houseless [9]. Regardless of the timing of care, patients who are houseless perceive high levels of stigma among healthcare providers [10, 11]. This stigma often leads to mistrust of healthcare providers, particularly for patients who also use substances [12]. Experiences that stigmatize substance use disorder care may further drive these patients from care [13]. These interactions are driven not only by individual providers, but also by healthcare policies created and implemented at a systems-level. For example, McNeil et al. described how healthcare system policies around in-hospital substance use, combined with undertreated pain and withdrawal, contributes to patients leaving hospitals against medical advice [13]. Stigmatizing care can actively harm patients who critically need access to equitable healthcare [14, 15].

Stigmatizing systems are not unchangeable. Healthcare systems have the ability to change institutional policies to structurally improve care (for example, the creation of addiction medicine consult services to support the care of patients with addiction [16]). They also often hold tremendous power in their state and local governments. However, to change institution, state and federal level policies to improve care for people who are houseless, institutions must understand the needs and perspectives of houseless community members.

Medical students and medical schools have historically advocated to improve healthcare systems that care for people who are houseless. There are three unique aspects of being a medical student that allow students to be effective advocates in this area: 1) the ability to hold their institutions accountable by calling out systemic injustices, 2) the opportunity to seek diverse clinical training and learn from experts, and 3) ample time to participate in work with people who are underserved, particularly prior to the clinical phase of medical training. Extensive research from medical schools and students across the country has sought to take advantage of each of these role-characteristics to improve care for people who are homeless within institutions. This research focused primarily on improving attitudes and knowledge around houselessness among medical students [17–22]; altering didactic medical education to include training on caring for people who are houseless or otherwise excluded from care [20, 23–26]; facilitating opportunities to care for people who are houseless in clinical settings [25, 27–29]; and more broadly improved the social accountability of medical schools and the institutions they are a part of [30–33]. These efforts are significant not only for enacting change at the institutional level, but also for preparing the next generation of physicians to provide full-spectrum and empathic care to people who are houseless.

While healthcare systems may struggle to adequately care for people with addiction and mental health conditions, medical students and the schools that train them have historically had unique and important roles in improving care for these populations. By teaching students how to better care for marginalized patients and by students pressuring institutions to make changes that support these patients, institutions can and have pursued change at not only the hospital level, but also at state and federal policy levels. However, little research has identified exactly how people who are houseless would like medical students to wield this potential power. Changes in policies that impact people who are houseless must involve them directly. The objective of this study is to understand perceptions of how medical students and institutions can meet needs of the houseless community.

## Methods

### Study site

Within Portland, Oregon city limits, there are over 4000 people who are houseless or marginally housed individuals per night; the city has a population of just over 647,000 people [34]. Oregon Health & Science University (OHSU) works to serve people who are houseless through collaborations with Central City Concern, a large non-profit medical home for people who are low-income or houseless in the city, though students are not routinely involved in care delivery settings through this clinic. OHSU itself is located on top of a hill in Portland; most people going to the hospital either take an air-tram from the base of the hill to the hospital or drive or bus to the top. OHSU School of Medicine offers an 8-week continuity elective at Central City Concern that is available to a very limited number of students. Students at OHSU also operate an interprofessional free clinic in partnership with Transition Projects, a shelter and community-based organization, and may volunteer or receive credit for their involvement. While there are limited opportunities for student engagement through OHSU, there are many community-based organizations in Portland that provide extensive support services to houseless community members, the majority of which are not affiliated with OHSU. For this reason, a decision was made to choose study sites in community spaces outside the OHSU network.

This study was completed by medical students at OHSU in Portland, Oregon (CK, CF, AC, JJ). OHSU medical students began collaborating with people who are houseless and the organizations that work to serve them in 2017. This collaboration led to the formation of two advisory groups: the Houseless Neighbor Advisory Team (HNAT), comprised of current, formerly houseless, or marginally housed individuals; and the Community Partner Advisory Team (CPAT), comprised of community partners and medical providers. These two research panels directly oversaw the research described in this paper. Both HNAT and CPAT meet with students to provide feedback at each step of the research process.

### Study design and setting

This mixed-methods study took place at two community locations in Portland, Oregon, during the winter and spring of 2018. With community input, we developed an interview guide that included both discrete choice (quantitative) and open-ended (qualitative) questions (Additional file 1), with the goal of understanding how medical students and institutions can serve people who are houseless.

Two sampling locations for interviewing people who are houseless were chosen in collaboration with HNAT and CPAT. The first location was Street Roots (SR), a community-run newspaper and media organization that allows people who are houseless to generate income through newspaper sales [35] . Street Roots is located in Portland’s Old Town/Chinatown neighborhood, and employs over 160 vendors. Medical students partnered with the Vendor Coordinator to identify if there was potential interest in having medical students talk to vendors visiting the site during morning coffee hours. Medical students interviewed participants on Saturday mornings from 7:30 am–9 am weekly.

The second data-collection location was Operation Nightwatch (ONW), a community-based hospitality center for people who are houseless that runs out of a local church on weekends [36]. Medical students worked with two Volunteer Coordinators (as one employee left, and another was hired) to identify if it was possible and appropriate to recruit study participants at ONW. Unlike SR, whose coffee hours were open exclusively to Street Roots Vendors, ONW is open to any guest visiting while open. Medical students interviewed participants at ONW on Saturday evenings from 7 to 10 pm.

We approached all individuals for consent until we had interviewed no more than half of our total sample goal (20 per site) in total. As medical students ourselves, we also wanted to understand how engagement that prioritizes community voices within our role as students might look. Participants were offered a coffee voucher and/or a snack at the beginning of the interview, and they were permitted to complete the interview alone or in small groups of two to three participants, if desired.

### Participants

Study participants were eligible to participate if they were 18, considered themselves houseless or marginally housed, and were English speaking. Our outreach teams, comprised of two to four medical students, approached and enrolled potential participants at the two sites (SR and ONW), and conducted the interviews.

### Study sample

We completed 38 interviews total at both ONW (*n* = 20) and SR (*n* = 18). This sample size was determined a priori in consultation with mixed-methods researchers at OHSU.

### Variables

#### Quantitative

The interview guide included questions adapted from the 2016 Seattle, Washington Houseless Needs Assessment [37] . A list of discrete-choice questions was presented to CNAT and HPAT in January of 2018. We used a mixed-methods approach at the suggestion of the panels, who asked us to incorporate both quantitative and qualitative questions to answer our research question. These questions were modified based on feedback from both panels.

Binary and categorical covariates include gender, sexual preference, race/ethnicity, highest education level, employment status, primary language, born in the United States, veteran status, types of places of residence in past month, history of using alcohol and drugs, history of comorbid conditions, comorbid conditions contribute to houselessness, history of receiving healthcare from provider in last year, interest in foot care information, interest in naturopathic medicine information, access to nutritious meals, history of pregnancy and current pregnancy status, interest in receiving information about pregnancy, access to clean needles, frequency of clean needle use, length of time houseless in years, and age at first time of houselessness. Our only continuous variable was age.

#### Qualitative data

A set of open-ended questions was drafted based on feedback from initial conversations with CNAT and HPAT in fall 2017. These questions were reviewed by CNAT and HPAT in January 2018 and modified based on feedback. We explored ideas related to the experience of participants engaging with healthcare systems before, during and after clinic or hospital visits, which we believed may shape the asks of healthcare providers and systems. We then asked what medical students and separately, healthcare systems can do to meet the goals of people who are houseless. Qualitative data was not recorded at the request of community members during study-planning phases; medical students took notes during interviews by hand. The interview guide included both discrete choice and open-ended questions (Additional file 1) and data were collected at the same time, but we prioritized qualitative information from the survey in analysis for this paper.

### Data analysis

#### Primary analysis

Univariate analyses displaying frequencies and means describe the quantitative sample. Quantitative data was entered into REDCap; basic frequencies were analyzed using STATA 14. Qualitative data was analyzed by four medical students trained in qualitative methods using thematic analysis rooted in an inductive process [38]. First, all four students (three female, one male) reread interview notes and transcripts to familiarize themselves with the data. Using a subset of interviews, we coded all interviews, and included codes that were related to answering our research question. We met regularly to discuss codes, and upon completion of coding, to identify themes and group codes within these themes. We then reviewed all codes and quotes within the context of the themes we constructed, and reorganized until consensus was met. Data analyzed was stored in the web-based program Dedoose. Mixing of quantitative and qualitative results occurred during the interpretation phase using a convergence study design.

#### Study results

Upon study completion, study results were shared with CNAT and HPAT for feedback on the analysis and to continue discussions of future planning for community-driven outreach and advocacy.

#### Consent and ethical considerations

Study participants provided verbal consent to participate. This project was approved by Portland State University’s Institutional Review Board. We applied for a waiver of written consent for this population, as the study was of minimal risk to participants. Participants gave verbal consent before beginning the study.

## Results

**Fig. 1:**
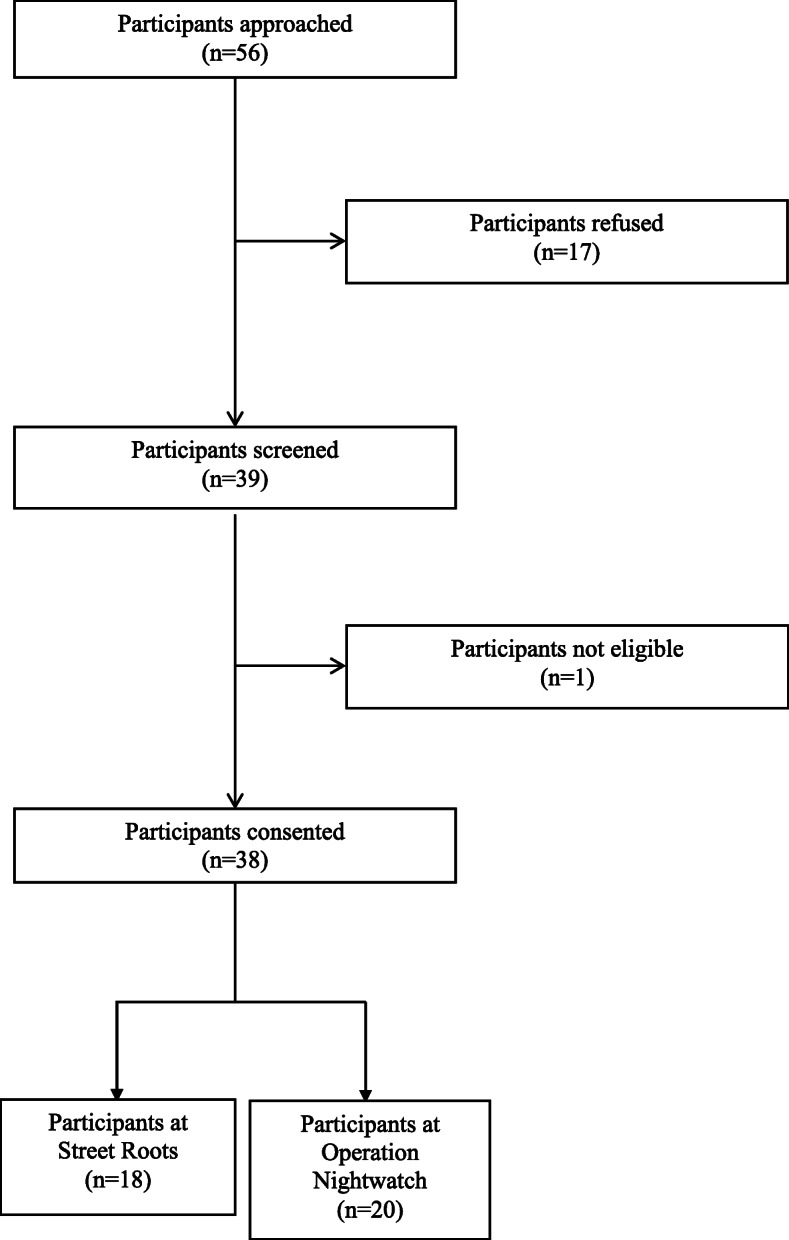
Enrollment flowchart from a study of community-derived recommendations for healthcare systems and medical students to support people who are houseless in Portland, Oregon, 2018

Over four visits to SR and one visit to ONW, we approached 56 individuals for consent; 17 people declined to participate, and 1 person screened was not eligible due to housing status (Fig. 1). We enrolled 38 total participants, 20 from ONW and 18 from SR (Fig. 1). Across both sites, most participants were male (73.7%), white (78.9%), and had been houseless for over a year at the time of interview (65.8%) (Table 1). We identified two qualitative themes in this work: 1) Care experiences among people with mental health and substance use disorders, and 2) Roles for medical students and health-care institutions.

**Table 1:** Participant demographics from a study of community-derived recommendations for healthcare systems and medical students to support people who are houseless in Portland, Oregon, 2018

	Street Roots (***n*** = 18)	ONW (***n*** = 20)	Total (***n*** = 38)
**Age**	51.0 (11.1)	47.6 (13.9)	49.1 (12.6)
**Gender** Male	14 (77.8%)	14 (70.0%)	28 (73.7%)
**Transgender**	1 (5.6%)	2 (10.0%)	3 (7.9%)
**LGBQ**	2 (11.1%)	6 (30.0%)	8 (21.1%)
**Race** White	13 (72.2%)	17 (85.0%)	30 (78.9%)
Asian	0	0	0
Native Hawaiian Pacific Islander	0	1 (5.0%)	1 (2.6%)
Black/African American	2 (11.1%)	1 (5.0%)	3 (7.9%)
AI/AN	1 (5.6%)	1 (5.0%)	2 (5.3%)
Hispanic	0	0	0
**English primary language**	18 (100%)	19 (95.0%)	37 (97.4%)
**Born in US**	17 (94.4%)	18 (90.0%)	35 (92.1%)
Missing	0	1 (5.0%)	1 (2.6%)
**Veteran**	2 (11.1%)	4 (20.0%)	6 (15.8%)
**Highest Education**			
< 12th grade	4 (22.2%)	4 (20.0%)	8 (21.1%)
High school/GED	4 (22.2%)	5 (25.0%)	9 (23.7%)
Some college/associates	4 (22.2%)	10 (50.0%)	14 (36.8%)
College degree	5 (27.8%)	0	5 (13.2%)
Graduate degree	0	1 (5.0%)	1 (2.6%)
Missing	1 (5.6%)	0	1 (2.6%)
**Currently unhoused**			
7 days or less	1 (5.6%)	1 (5.0%)	2 (5.3%)
8–30 days	0	2 (10.0%)	2 (5.3%)
1–3 months	1 (5.6%)	2 (10.0%)	3 (7.9%)
4–6 months	0	1 (5.0%)	1 (2.6%)
7–11 months	0	0	0
1 year	0	2 (10.0%)	2 (5.3%)
More than 1 year	16 (88.9%)	9 (45.0%)	25 (65.8%)
Missing	0	3 (15.0%)	3 (7.9%)
**Age of first homeless**			
0–17 years	6 (33.3%)	6 (30.0%)	12 (31.6%)
18–24 years	3 (16.7%)	5 (25.0%)	8 (21.1%)
25–35 years	2 (11.1%)	3 (15.0%)	5 (13.2%)
36–49 years	3 (16.7%)	2 (10.0%)	5 (13.2%)
50–65 years	3 (16.7%)	1 (5.0%)	4 (10.5%)
> 66 years	1 (5.6%)	1 (5.0%)	2 (5.3%)
Missing	0	2 (10.0%)	2 (5.3%)
**Longest consecutive unhoused**			
7 days or less	0	0	0
8–30 days	0	0	0
1–3 months	0	0	0
4–6 months	0	3 (15.0%)	3 (7.9%)
7–11 months	1 (5.6%)	1 (5.0%)	2 (5.3%)
1 year	0	1 (5.0%)	1 (2.6%)
More than 1 year	17 (94.4%)	14 (70.0%)	31 (81.6%)
Missing	0	1 (5.0%)	1 (2.6%)
**Spent a night in last month**			
Outdoor location	7 (38.9%)	11 (55.0%)	18 (47.4%)
Friend’s house	3 (16.7%)	1 (5.0%)	4 (10.5%)
Family member’s home	1 (5.6%)	2 (10.0%)	3 (7.9%)
Squat/abandoned building	1 (5.6%)	0	1 (2.6%)
Public facility	5 (27.8%)	0	5 (13.2%)
Motel room	2 (11.1%)	0	2 (5.3%)
Car	3 (16.7%)	0	3 (7.9%)
Train car	1 (5.6%)	0	1 (2.6%)
Other	1 (5.6%)	0	1 (2.6%)
**Case worker**	4 (22.2%)	6 (30.0%)	10 (26.3%)
**Health Insurance**	17 (94.4%)	17 (85.0%)	34 (89.5%)
Missing	0	1 (5.0%)	1 (2.6%)
**Health insurance type**			
OHP	10 (55.6%)	12 (60.0%)	22 (57.9%)
Other Medicaid	3 (16.7%)	0	3 (7.9%)
Medicare	3 (16.7%)	2 (10.0%)	5 (13.2%)
Private insurance	0	0	0
Other	1 (5.6%)	2 (10.0%)	3 (7.9%)
Missing	0	0	1 (2.6%)
**Substances**			
Alcohol	5 (27.8%)	5 (25.0%)	10 (26.3%)
Methamphetamine	3 (16.7%)	3 (15.0%)	6 (15.8%)
Heroin	3 (16.7%)	0	3 (7.9%)
Crack	0	0	0
Cocaine	1 (5.6%)	0	1 (2.6%)
Cannabis	14 (77.8%)	5 (25.0%)	19 (50.0%)
Fentanyl	1 (5.6%)	0	1 (2.6%)
Other drug	4 (22.2%)	0	4 (10.5%)
No drug use	3 (16.7%)	9 (45.0%)	12 (31.6%)
**Clean needle access**			
Yes	6 (33.3%)	4 (20.0%)	10 (26.3%)
No	0	0	0
Not applicable	12 (66.7%)	16 (80.0%)	28 (73.7%)
**Clean needle use**			
Yes	3 (16.7%)	2 (10.0%)	5 (13.2%)
Sometimes	3 (16.7%)	1 (5.0%)	4 (10.5%)
No	0	0	0
Not applicable	12 (66.7%)	17 (85.0%)	29 (76.3%)
**Health conditions**			
Diabetes Mellitus	1 (5.6%)	4 (20.0%)	5 (13.2%)
Cancer	1 (5.6%)	0	1 (2.6%)
PTSD	10 (55.6%)	7 (35.0%)	17 (44.7%)
Bipolar	5 (27.8%)	1 (5.0%)	6 (15.8%)
Depression	10 (55.6%)	7 (35.0%)	17 (44.7%)
Schizophrenia	1 (5.6%)	1 (5.0%)	2 (5.3%)
Other psych	9 (50.0%)	6 (30.0%)	15 (39.5%)
Physical disability	4 (22.2%)	1 (5.0%)	5 (13.2%)
TBI	1 (5.6%)	0	1 (2.6%)
Liver disease	2 (11.1%)	0	2 (5.3%)
Other STI	1 (5.6%)	0	1 (2.6%)
PTSD clarification	9 (50.0%)	7 (35.0%)	16 (42.1%)
**Pregnant in last 5 years (*n*** **= 10)**	0	1 (10.0%)	1 (10%)
**Interested in pregnancy learning (*n*** **= 10)**			
Maybe	0	2 (20.0%)	2 (20%)
No	18 (100%)	18 (90.0%)	36 (94.7%)
**Medical conditions prevent housing**			
Yes	9 (50.0%)	9 (45.0%)	18 (47.4%)
Missing	1 (5.6%)	2 (10.0%)	3 (7.9%)
**Healthcare seeking**			
Free clinic	0	0	0
Outside In	5 (27.8%)	1 (5.0%)	6 (15.8%)
Central City Concern	5 (27.8%)	1 (5.0%)	6 (15.8%)
Legacy Emanuel	6 (33.3%)	4 (20.0%)	10 (26.3%)
Legacy Good Samaritan	7 (38.9%)	5 (25.0%)	12 (31.6%)
Providence Portland Med Center	3 (16.7%)	1 (5.0%)	4 (10.5%)
Adventist Med Center	1 (5.6%)	0	1 (2.6%)
Portland Shriners Hospital	0	0	0
OHSU	6 (33.3%)	2 (10.0%)	8 (21.1%)
Urgent care	1 (5.6%)	1 (5.0%)	2 (5.3%)
Private Doctor	2 (11.1%)	1 (5.0%)	3 (7.9%)
VA	0	2 (10.0%)	2 (5.3%)
Planned Parenthood	0	0	0
Wallace Medical Concern	0	0	0
Richmond Clinic	2 (11.1%)	0	2 (5.3%)
Other	4 (22.2%)	2 (10.0%)	6 (15.8%)
No care	1 (5.6%)	6 (30.0%)	7 (18.4%)
**Interested in naturopathic learning**			
Yes	14 (77.8%)	8 (40.0%)	22 (57.9%)
Maybe	2 (11.1%)	4 (20.0%)	6 (15.8%)
Missing	0	1 (5.0%)	1 (2.6%)
**Interested in foot care learning**			
Yes	8 (44.4%)	9 (45.0%)	17 (44.7%)
Maybe	2 (11.1%)	1 (5.0%)	3 (7.9%)
Missing	0	2 (10.0%)	2 (5.3%)
**Access to one healthy meal/day**			
Yes	17 (94.4%)	16 (80.0%)	33 (26.8%)
Maybe	1 (5.6%)	1 (5.0%)	2 (5.3%)
Missing	0	1 (5.0%)	1 (2.6%)

### Theme 1: care experiences among people with mental health and substance use disorders

Experiences seeking care and perceptions of healthcare systems and students were largely described in the context of seeking care as people with mental health conditions, substance use disorders, or both. Participants experiences as people with substance use disorder or mental health conditions shaped their perspective of how medical students and institutions should help support people who are houseless.

#### Substance use

Half of participants reported cannabis use, while 31.6% stated they do not use drugs or drink alcohol. Patients who use drugs or alcohol expressed frustration working with doctors and hospital staff. Many participants that used drugs felt judged by their providers. “Some doctors are fucking dicks, [they] don’t know anything about being a drug addict.”-Participant 28. Another participant was told he could not be prescribed Xanax while taking methadone, so he would use his own money to buy Xanax on the streets while adhering to his methadone schedule. This participant was frustrated that the system would not allow him to access the medications he felt he needed for his mental health condition.

Another patient stated that he should, “be able to get a prescription for meth. Addicts should get prescriptions from a doctor and go to safe using sites and use. Addiction is an illness. What the hell is people’s problems in the US?” -Participant 28. Additionally, several patients identified drug addiction as a medical condition they are currently managing that they feel prevents them from maintaining control of their life. Participant 16 succinctly stated, “addiction always draws you back in.”

Importantly, participants shared that stigmatizing care around their substance use disorders was a barrier to engagement. One participant described simply falling out of care as his methamphetamine use disorder worsened. He and his husband are both HIV positive, and described challenges identifying how to engage in HIV care in Portland, which he felt was confounded by his and his husband’s use of methamphetamine.

Many participants felt that when presenting to emergency departments, they had to work to convince people that they were not seeking drugs and that their pain was real. One participant said, “I was beat up by a cop and had a black eye, [and I] got a letter in the mail that said, ‘Please do not come to our clinic unless you have an emergency’.” -Participant 30. He felt that providers did not believe he was injured. Participant 7 said that they went to the emergency department with a sprained ankle and history of alcoholism but they “don’t believe people,” and instead assume that they are drug-seeking.

#### Mental health

Nearly half of participants shared that they had been diagnosed with Post-Traumatic Stress Disorder (PTSD) (44.7%), and an additional two-fifths (39.5%) of participants identified that they had a psychiatric condition other than PTSD. Participants identified barriers to accessing mental health care, including long wait times to get in to see providers and accessing services for non-emergency mental health. One participant stated that they had been diagnosed with Bipolar Disorder and needed care. At the time of the survey, they had been in treatment for 6 months:

*“[It was] three years until I found a doctor. Long wait lists and specific call times [were barriers].” – Participant 1.*

Other participants stated that working closely with case managers, mental health providers and physicians had helped them better understand and manage their mental health care. A common theme among these participants highlighted the relationship building and need for longitudinal partnerships in providing useful mental health care. One participant discussed his in-and-out history of mental health care in Portland, and how medication had helped him after working with a medical team for a long time.

*“I take my ‘coo coo cookies’ and I’m alright. I work very closely with [the medical team], not against them. I highly recommend taking something- (referring to psychiatrics medications) ain’t high grade anymore. You’ll feel better… there’s no shame in taking meds.” – Participant 11.*

Other participants reiterated the challenges accessing non-emergent, non-judgemental, affordable mental health care.

### Theme 2: roles for medical students and institutions

#### Medical students

Participants were asked what medical students and OHSU (separately) could do to help support people who are houseless. Experiences in addiction care largely shaped participants asks of medical students. Participants asked that said that students learn to meet people where they are, learn about addiction, and be willing to engage with folks who are houseless without stigmatizing them. A participant noted that to do this, students must be willing to hear from people with lived experiences with houselessness, saying, “For one thing, you listen.” -Participant 12. One participant said that medical students should “learn to keep an open mind with people who use drugs.”-Participant 15. Multiple participants asked that students participate in rotations at organizations that primarily treat people who are houseless. One participant said students must “continue to get more exposure to treat people better in clinical settings.” -Participant 22. Another said that medical students shouldn’t “mold people” and should instead “serve people.”-Participant 37. Across the board, participants talked about destigmatizing houselessness, substance use, and mental health, and that learning these skills in medical school can help create better physicians down the road.

#### Institutions

Multiple participants asked OHSU to lobby on their behalf to change laws that criminalize drugs and houselessness. Participants believe that OHSU should “lobby Congress, get involved in City Hall, politically involved, advertise needs for the general community. OHSU is a big voice in this community.” -Participant 28. Participants also named city officials that they would like OHSU to partner with. Where OHSU cannot lobby, participants said that OHSU should do the right thing and do what is in the best interest of their patients, regardless of the consequences. One participant said, “If you have the ability to help someone, you should ignore the law and help them. Morally.” -Participant 3. Others noted the recent local conversations around safe injection sites, asking OHSU to take a prominent stand on this topic. “[We need] safe spots to shoot up.”-Participant 4.

Finally, participants directly asked OHSU to help support building better systems of care around addiction and mental health, saying that OHSU should “help people with addiction”- Participant 36. Participant 25 said, “Look around you, we put people in jail for things they used to put you in the hospital for. My generation put you in a hospital, in yours they put you in jail…. [OHSU should] fix the broken mental health system.”

## Discussion

In this study of 38 people currently houseless, we sought to identify avenues through which medical students and institutions can meet the needs of the houseless community. Participants in this study were majority white, male, currently houseless, and houseless for greater than 1 year. We identified two qualitative themes: care experiences among people with mental health and substance use disorders, and roles for medical students and health-care institutions.

The experiences of participants who had sought care for substance use disorders and mental health conditions, shaped their understanding of how institutions and medical students can help serve them. Participants noted that providers they have engaged with have a lack of knowledge about addiction, harbor stigma, and do not believe their pain or their stories. In terms of mental health care, people stated that they could not access care, and that they need to be able to find and access care that builds longitudinal relationships in order to feel well.

These experiences build on significant qualitative literature which highlights the mistrust of people with addiction in healthcare systems. Pain management is a challenging topic for providers in hospital settings, particularly with patients who may have addiction [39]. McNeil et al. described the social and structural factors in hospital settings that can lead to inadequate pain management, and subsequent breaks in trust with people with substance use disorders [13]. Our work builds on this to describe ways in which medical students and institutions can mitigate further harm from healthcare institutions, while grounding these efforts in the self-identified needs and lived experiences of community members.

Our second qualitative theme identified roles for medical students and healthcare institutions to better care for people who are houseless. Participant asks were largely rooted in changes that would improve care for substance use disorders and mental health care. Participants asked both medical students and institutions to work to improve healthcare systems and healthcare experiences for them and members of their community. Community members noted that medical students can serve people who are houseless, have substance use disorders, and/or have mental health conditions by working to: 1) listen to and believe people in their communities and in clinics, 2) destigmatize houselessness, addiction and mental health conditions among their peers and within the systems they are a part of, 3) engage in diverse clinical experiences, and 4) advocate that healthcare systems better care for people, in the ways they request.

Healthcare institutions have much work to do to help support people are houseless, but importantly, also have the power to make substantial change. First, community members want healthcare institutions to lobby to change laws that criminalize substance use and houselessness. Second, people want access to ways to keep themselves safe, in particular, safe injection sites, regardless of legal consequences; people who are houseless see the manifestation of safe injection sites created by hospitals as a moral imperative. This is rooted in the community’s third ask: that hospital systems act in the best interests of all of their patients, regardless of their struggles with addiction, mental health conditions, or houselessness. Finally, participants want healthcare systems to actively build better systems to care for them, particularly around addiction, mental health, and houselessness.

As previously mentioned, healthcare systems have the power to change. The bidirectional relationships between medical students and the institutions they are part of suggest that medical students can help support change for people who are houseless at the institutional (and state and federal) levels, and that healthcare institutions can likewise support the training and education of medical students to better care for people who are houseless in the future. This research builds on past work to understand how medical students and the institutions they are a part of can better care for people who are houseless, including through the development of socially-conscious medical schools as a whole [30–33]. Medical students have pushed to improve didactic [20, 23–26] and clinical [25, 27–29] opportunities to care for people who are houseless. These programs have helped support the professional development and education of other medical students, but have not always given space to community members to make their needs, preferences, and experiences heard. This work seeks to provide this space so that the voices of people who are houseless can in turn encourage medical students to advocate for change, both as trainees and future physicians, and help hold healthcare systems accountable for meeting the needs of this population.

This project had several strengths, including the research team’s partnership with community members, key stakeholders and organizations that serve people who are houseless in Portland. Specifically, community members had opportunities to provide critical feedback on this work throughout all phases of the study; feedback from both CPAT and HNAT was incorporated into this work. While brief, this study design, including both quantitative and qualitative questions in one interview guide, allowed the research team to better capture the complexity of people’s interactions with healthcare and with their surroundings.

This study was limited by the use of hand-written notes versus formal recording and transcription for qualitative work. Additionally, while we had many discussions with CPAT, HNAT and others about community locations to best to conduct this study, we may have missed critical voices by limiting our sample size to 40 participants at only two sites. Finally, by surveying at community-based organizations, we may have missed younger people who do not use these services and people who are more recently houseless. Future work should investigate how to best incorporate the voices of other people who are houseless into changes in healthcare settings.

## Conclusion

This study highlights the voices of people in Portland, Oregon who are houseless. Outcomes from this research will be used to guide future programming in partnership with OHSU medical students. Our research team believes in the principle of “nothing about us without us,” and we hope to continue to realize this in future planning for work between medical students and people who are houseless. Specifically, at our institution and others, medical student engagement with people who are houseless should be informed by the perceived needs of the community, and healthcare institutions must work to listen and support the marginalized members of their communities.

## Supplementary information

**Additional file 1: Appendix 1.** Interview Guide, Interview guide from study GRAMMS Checklist, Completed GRAMMS Checklist for mixed-methods study.

## Data Availability

The datasets generated during the study are not publicly available due to the sensitive nature of the data collected, but are available from the corresponding author on reasonable request and pending approval to share from Portland State University’s IRB.

## Abbreviations

OHSU: Oregon health and science university
CPAT: Community partner advisory team
HNAT: Homeless neighbor advisory team
SR: Street roots
ONW: Operation nightwatch
PTSD: Post traumatic stress disorder
