# Supplementary material for: Community-derived recommendations for healthcare systems and medical students to support people who are houseless in Portland, Oregon: a mixed-methods study
Source: BMC Public Health. 2020 Sep 2;20:1337. doi: 10.1186/s12889-020-09444-4 (PMC7466795; doi:10.1186/s12889-020-09444-4)
Supplement: Supplementary file 1 — Additional file 1: Appendix 1. Interview Guide, Interview guide from study GRAMMS Checklist, Completed GRAMMS Checklist for mixed-methods study. [file 12889_2020_9444_MOESM1_ESM.docx]

**Appendix 1. Semi-structured Qualitative Interview Guide**

1. Do you have an illness or disability that needs treatment or is being treated? [Probe: Can you tell me a little about that?]
2. What do you do if you are sick or injured? [Probe: Tell me about a time you were sick or injured and had to seek medical care; what if one of your friends/family becomes sick or injured?]
3. What are your biggest challenges to staying or getting healthy? [Probe: sleep, medication, exposure to weather, lack of safety]
4. Do you feel like you're in control of your health? [Probe: tell me a little about how you stay health]
5. What do you think medical students could do to help support your health?
6. What do you think OHSU could do to help support your health?
7. Other notes from interviewer
